# Anti-Tuberculosis Activity of Three Carbapenems, Clofazimine and Nitazoxanide Using a Novel Ex Vivo Phenotypic Drug Susceptibility Model of Human Tuberculosis

**DOI:** 10.3390/antibiotics11101274

**Published:** 2022-09-20

**Authors:** Ximena Gonzalo, Magdalena K. Bielecka, Liku Tezera, Paul Elkington, Francis Drobniewski

**Affiliations:** 1 Department of Infectious Diseases, Faculty of Medicine, Imperial College, London W12 0NN, UK; 2 NIHR Respiratory Biomedical Research Unit, Clinical and Experimental Sciences Academic Unit, Faculty of Medicine, University of Southampton, Southampton SO16 6YD, UK

**Keywords:** three-dimensional bioelectrospray, tuberculosis, anti-microbial drug resistance, drug susceptibility testing

## Abstract

We evaluated a novel physiological 3-D bioelectrospray model of the tuberculosis (TB) granuloma to test the activity of a known anti-TB drug, clofazimine; three carbapenems with potential activity, including one currently used in therapy; and nitazoxanide, an anti-parasitic compound with possible TB activity (all chosen as conventional drug susceptibility was problematical). PBMCs collected from healthy donors were isolated and infected with *M. tuberculosis* H37Rv lux (i.e., luciferase). Microspheres were generated with the infected cells; the anti-microbial compounds were added and bacterial luminescence was monitored for at least 21 days. Clavulanate was added to each carbapenem to inhibit beta-lactamases. *M. tuberculosis* (MTB) killing efficacy was dose dependent. Clofazimine was the most effective drug inhibiting MTB growth at 2 mg/L with good killing activity at both concentrations tested. It was the only drug that killed bacteria at the lowest concentration tested. Carbapenems showed modest initial activity that was lost at around day 10 of incubation and clavulanate did not increase killing activity. Of the carbapenems tested, tebipenem was the most efficient in killing MTB, albeit at a high concentration. Nitazoxanide was effective only at concentrations not achievable with current dosing (although this might partly have been an artefact related to extensive protein binding).

## 1. Introduction

Tuberculosis (TB) is a major killer, representing the main infectious cause of mortality in the world until 2020. There are over 10 million new cases every year with approximately 1.5 million deaths in 2019 [1].

Repurposing drugs to use against multi-drug-resistant (MDR-TB) and extensively-drug-resistant tuberculosis (XDR-TB) has become a strategy in the search for active compounds against the most resistant forms of the disease. Several families of drugs have been investigated, including anti-microbials and non-anti-infective drugs [2].

Most of the information about these compounds comes from in vitro studies that are devoid of host cells, or animal models that do not replicate the role of human cells in both the pathogenicity and the response to anti-microbial therapy. The aim of this work was to test the activity of several drugs against *M. tuberculosis* (MTB) H37Rv in a 3-D bioelectrospray system containing cells from human donors and an extracellular matrix component, a more physiological model of the granuloma [3,4,5]. For example, pyrazinamide susceptibility is difficult to assay accurately using conventional laboratory microbiology cultures but we have previously demonstrated pyrazinamide susceptibility correctly in this system, showing that it replicates the correct physiological environment seen in patients [4].

The drugs chosen were (1) clofazimine, a compound in use for many decades against *M. leprae* and now in use against MTB and whose relevance has been highlighted by the development of the short MDR-TB treatment course and the new all-oral regimen [6,7]; (2) carbapenems, beta-lactam broad spectrum anti-bacterials active against a wide range of Gram-positive and Gram-negative microorganisms that have been used against mycobacteria and MTB for over a decade [8]; and (3) nitazoxanide, an anti-parasitic drug that has been found to have anti-MTB activity in vitro [9,10]. In addition to the extensive track record of use in humans with good tolerance, the antibiotics used in the study are not toxic to human cells at the concentrations studied [11,12,13,14]. Furthermore, in previous studies, where we formally checked toxicity, no toxic antibiotic effect on PBMCs was seen ([4] Figure S5).

## 2. Results

A full set of luminescence results is available in Supplementary Table S1. We did not perform matching CFU studies as we have previously shown excellent correlation between luminescence and CFU [4].

**Figure 1:**
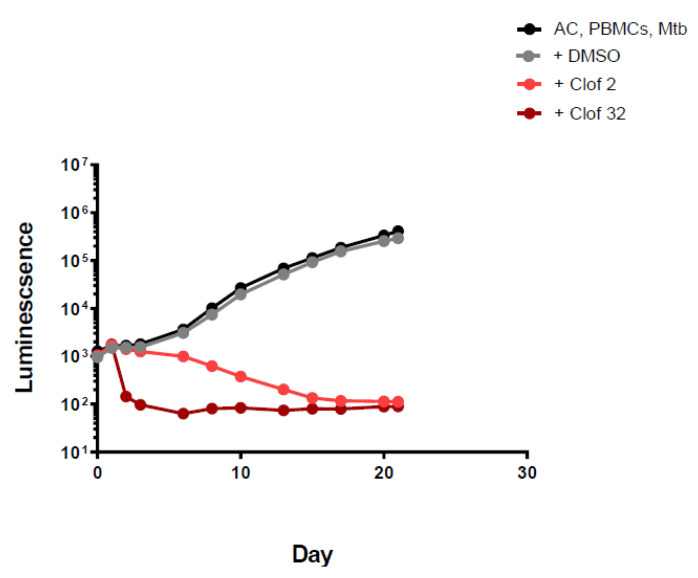
Luminescence detection at different time points for clofazimine in TB-infected PMBCs obtained from donor 1. AC: alginate collagen; PMBCs: peripheral blood mononuclear cells; Mtb: Mycobacterium tuberculosis; DMSO: dimethyl sulfoxide; Clof 2: Clofazimine 2 mg/L; Clof 32: Clofazimine 32 mg/L.

Clofazimine showed good killing activity at both concentrations tested and was the only drug of the panel that killed bacteria at the lowest concentration tested (which is within that obtainable with standard dosing). At 2 mg/L, in both experiments, it showed good activity from the beginning, achieving a 1 log reduction in luminescence by day 20, with no change after that time point (only experiment 2) Figure 1 and Supplementary Figure S1.

**Figure 2:**
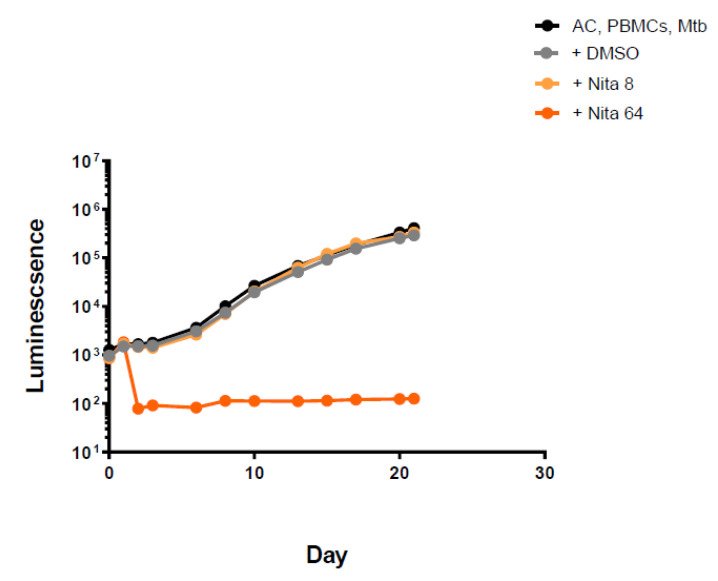
Luminescence detection at different time points for nitazoxanide in TB-infected PMBCs obtained from donor 1. AC: alginate collagen; PMBCs: peripheral blood mononuclear cells; Mtb: Mycobacterium tuberculosis; DMSO: dimethyl sulfoxide; Nita8: Nitazoxanide 8 mg/L; Nita 64: Nitazoxanide 64 mg/L.

Nitazoxanide was effective only at the highest concentration tested, showing no activity at 8 mg/L (Figure 2 and Supplementary Figure S2).

**Figure 3:**
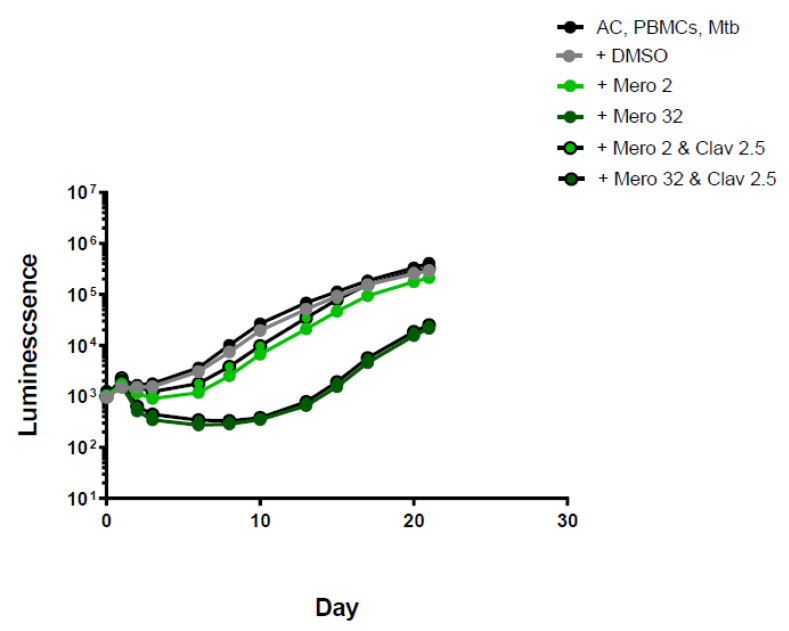
Luminescence detection at different time points for meropenem in TB-infected PMBCs obtained from donor 1. AC: alginate collagen; PMBCs: peripheral blood mononuclear cells; Mtb: Mycobacterium tuberculosis; DMSO: dimethyl sulfoxide; Mero 2: Meropenem 2 mg/L; Mero 32: Meropenem 32 mg/L; Clav 2.5: clavulanic acid 2.5 mg/L.

Carbapenems showed some modest initial activity that was lost at around day 10 of incubation. Meropenem, the model drug for the carbapenem group, showed good inhibition at a concentration of 32 mg/L up to the tenth day of incubation. Bacterial growth increased after day 10, suggesting the meropenem either only exerted a bacteriostatic effect and/or degradation of the drug occurred in the medium after a few days. The addition of clavulanic acid exerted a modest effect on meropenem activity but did not produce any differences in luminescence for the remaining carbapenems (Figure 3 and Supplementary Figure S3).

**Figure 4:**
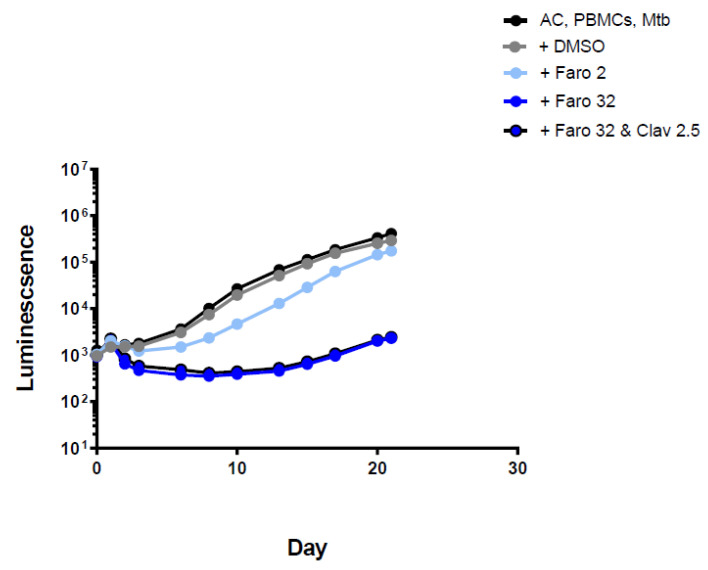
Luminescence detection at different time points for faropenem in TB-infected PMBCs obtained from donor 1. AC: alginate collagen; PMBCs: peripheral blood mononuclear cells; Mtb: Mycobacterium tuberculosis; DMSO: dimethyl sulfoxide; Faro 2: Faropenem 2 mg/L; Faro 32: Faropenem 32 mg/L; Clav 2.5: clavulanic acid 2.5 mg/L. Standard deviation included but are very small, i.e., within the size of the points on the curve.

Faropenem showed a similar result except that luminescence only started to increase later, at around day 15. Addition of clavulanate did not improve the killing effect (Figure 4 and Supplementary Figure S4).

**Figure 5:**
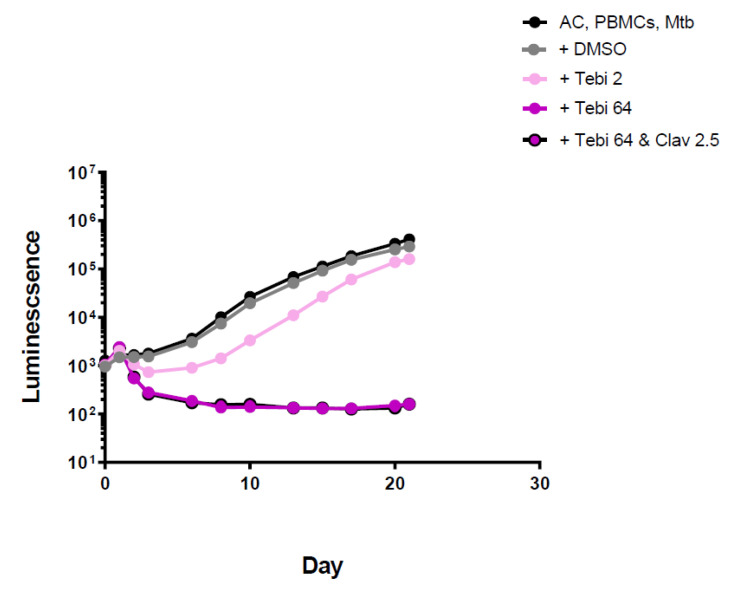
Luminescence detection at different time points for tebipenem in TB-infected PMBCs obtained from donor 1. AC: alginate collagen; PMBCs: peripheral blood mononuclear cells; Mtb: Mycobacterium tuberculosis; DMSO: dimethyl sulfoxide; Tebi 2: Tebipenem 2 mg/L; Tebi 64: Tebipenem 64 mg/L; Clav 2.5: clavulanic acid 2.5 mg/L.

Tebipenem was active at 64 mg/L. The luminescence curve remained stable after 10 days, suggesting it is more stable or active than the other two carbapenems in this model. Addition of clavulanate did not increase killing activity (Figure 5 and Supplementary Figure S5).

## 3. Discussion

Here, we study different anti-microbials in a 3-D granuloma model of human TB that has previously been shown to replicate pyrazinamide efficacy against TB [4]. Clofazimine, an anti-leprosy drug, has been known to be active against TB for several decades [15] and has been in use against TB (especially multi-drug-resistant TB (MDR-TB)) for many years now with mounting evidence about its effectiveness and safety profile [6,16,17,18]. It is now one of the standard core drugs for MDRTB therapy [7].

However, in vitro tests are challenging and reliable methods for testing of key drugs, (some of which have only recently been developed) [19], are not easily reproducible in resource-limited settings or are difficult to implement outside reference centres [20]. Reliable testing challenges even national reference laboratories [21]. The results obtained in these experiments offer additional evidence of the activity of this compound against TB and further evidence of the value of the 3-D system as a reliable alternative drug susceptibility assay that mimics human physiology better than conventional microbiological culture assays. The 3-D system, therefore, could present a gold standard for testing sensitivity, compared to other standard microbiological solid or broth culture methods [21].

Nitazoxanide has been tested in vitro against TB before with good results, with MICs around 16 mg/L [9]. However, in the 3-D model, it showed some activity but at concentrations not achievable in vivo with the current dosing regimen [22]. This drug binds extensively [9] to protein; hence, it is challenging to test and interpret in vitro or ex vivo experimental results as it is not clear whether the drug has limited activity or if it is attached to protein in the medium, and unable to reach the bacterial target. Further work should include testing the actual active metabolite in a model requiring low-protein media.

Carbapenems’ activity against mycobacteria has been reported extensively in the last two decades [23,24,25]. However, conflicting in vitro results, different methodologies and different drug choice have contributed to the lack of clarity regarding which carbapenem is the best for use in human disease and what method, if any, is the best for testing [24,25,26,27]. Clinical outcome evidence is limited and difficult to interpret as TB therapy of MDR and XDR-TB involves the combination of several drugs. Currently, no well-powered control trial exists [23]. Our experiments in a more physiological granuloma model show that tebipenem is more effective than the other carbapenems tested, but none of them showed activity at concentrations achievable in vivo with current recommended doses.

Analysis of antibiotic efficacy in a 3-D granuloma model complements findings in more standard systems by analysing drug efficacy in an environment with greater physiological similarity to the human host. For example, host gene expression more closely reflects that in patients compared to 2-D models [28], multinucleate giant cells form and T cells proliferate [3] and cells migrate to form granulomas [29]. Within this 3-D model, the *M. tuberculosis* killing efficacy of the compounds tested was shown to be dose-dependent.

Clofazimine was the most effective antibiotic tested. It inhibited the growth of *Mycobacterium tuberculosis* at as low as 2 mg/L in the 3-D bioelectrospray model.

Nitazoxanide requires further work to establish whether the lack of activity is a true drug-related phenomenon or if it is an artefact related to extensive protein binding. Additionally, the active compound, nitazoxanide metabolite tizoxanide, would be a better drug to test.

The role of carbapenems remains unclear and this work can offer limited clarification. Of the ones tested here, tebipenem was the most efficient in killing MTB, albeit at a high concentration of 64 mg/L. The addition of clavulanate did not increase their killing activity significantly.

A potential limitation of the 3-D biomimetic model is that it requires extensive infrastructure and expertise, and so steps to minimise the equipment and training needed are needed to increase accessibility and widespread uptake.

## 4. Materials and Methods

### 4.1. Peripheral Blood Mononuclear Cells (PBMCs) 

PBMCs were isolated from single-donor buffy coats from the UK National Health Service Blood and Transplant Service, Southampton, United Kingdom. Leukocytes were isolated by density gradient centrifugation over Ficoll-Paque (GE Healthcare Life Sciences, Chalfont St Giles, UK). The blood donors were from a region of the country with very low TB incidence, and were HIV and viral hepatitis B and C negative. Each experiment involved the use of PBMC taken from one donor and the experiment was performed in triplicate. A second donor then provided PBMC for the same experiment, which was again performed in triplicate 

### 4.2. Bacterial Culture

*M. tuberculosis* H37Rv (Mtb) was cultured in Middlebrook 7 H9 medium (supplemented with 10% ADC, 0.2% glycerol and 0.02% Tween 80) (BD Biosciences, Oxford, UK). Bioluminescent M. tuberculosis H37Rv lux [30] was cultured in 7H9 containing kanamycin (25 μg/mL) for all experiments. Cultures at 1 × 10^8^ CFU/mL Mtb (OD = 0.6) were used for all experiments at a multiplicity of infection (M.O.I) of 0.1.

### 4.3. PBMCs Encapsulation

Isolated PBMCs were infected with *M. tuberculosis* lux at a multiplicity of infection of 0.1 [4]. After overnight infection, infected cells were transferred from vented flasks to 50 mL Falcon tubes after detachment with Versene solution (Sigma Aldrich, Saint Louis, MO, USA) for 10 min and soft scraping, and then rinsed with Hanks’ balanced salt solution (HBSS) without Ca/Mg (Gibco, Jenks, OK, USA). The cells were centrifuged at 320× *g* for 8 min at 4 °C, and the supernatant was decanted. The pelleted cells were then resuspended in an appropriate volume of a matrix composed of alginate (1.5%, Pronova UP MVG alginate, Nova Matrix, Sandvika, Norway) with human collagen (1 mg/mL, Advanced BioMatrix, Carlsbad, CA, USA) at a final concentration of 5 × 10^6^ cells/mL.

The cell matrix was then encapsulated using an Electrostatic bead generator (Nisco Engineering, Zurich, Switzerland), following the protocol described in detail in Bio-protocol [5]. In brief, the cell-matrix mixture was transferred to a sterile syringe and injected into the bead generator at 10 mL/h via a Harvard syringe driver. Microspheres of 600 μm diameter formed when the matrix passed through a 0.7 mm external diameter nozzle, landing in a gelling bath of 100 mM CaCl_2_ in HBSS placed below the highly charged electrostatic ring that accelerated the microspheres from the needle head. Microspheres were collected from every 5 mL of matrix to 50 mL tubes and washed twice with HBSS with Ca/Mg and were transferred in RPMI 1640 medium containing 10% human AB serum and incubated at 37 °C, 21% O_2_ and 5% CO_2_. No media changes were performed, and the supernatant was harvested at defined time points for analysis. The specific drugs were added the next day as outlined below. Mtb growth within microspheres was monitored longitudinally by luminescence (GloMax 20/20 Luminometer, Promega, Southampton, UK) for at least 21 days.

### 4.4. Drugs and Concentration Tested

Drugs and concentrations tested are listed below: Meropenem (Sigma Aldrich, Saint Louis, MO, USA): 2, 8 and 32 mg/L; Faropenem (Sigma Aldrich, Saint Louis, MO, USA): 2, 8 and 32 mg/L; Tebipenem (MedChem Express, Princeton, NJ, USA): 2, 8 and 64 mg/L; Nitaxozanide (Sigma Aldrich, Saint Louis, MO, USA): 8 and 64 mg/L; Clofazimine (Sigma Aldrich, Saint Louis, MO, USA): 2 and 32 mg/L; Clavulanic acid (Sigma Aldrich, Saint Louis, MO, USA): 2.5 mg/L.

The concentrations were chosen as they contain the concentrations achievable in vivo with current dosing regimens, i.e., MEM 1 g, Cmax steady state: 14.14 ± 2.02 mg/L [31], TEB 400 mg Cmax 17,825 ± 5753 ng/mL [32], FAR 300 mg Cmax 14 mg/L [33], Clofazimine AUC day 14 4.2 mg/L and Clofazimine AUC 2 months 7.3 mg/L [34]. For MEM, trough concentrations (Cmin) of >64.2 mg/L and >44.5 mg/L were associated with neurotoxicity or nephrotoxicity, respectively [35]. For nitaxozanide, Cmax is 1.9 mg/L (range 1.1–2.5) 2–6 h after dosing, and an AUC is 3.9–11.3 mg∙h/L [36].

All drugs were dissolved in DMSO (except clavulanate, which was dissolved in distilled water). Stock solutions were filter-sterilised and frozen at −80 °C in 500 µL aliquots. Experiments were carried out in triplicate and performed on PBMCs from at least two separate donors on separate occasions. All drugs were dissolved in DMSO (except clavulanate, which was dissolved in distilled water). Stock solutions were filter-sterilised and frozen at −80 °C in 500 µL aliquots. Bacterial luminescence was monitored for at least 21 days. Standard deviations are included in Figures but were very small, i.e., within the size of the points on the curves.

## 5. Conclusions

Overall, analysis in a 3-D biomimetic model may help prioritise promising antibiotics, before they move forward to clinical trials, facilitating the development of new TB drug regimes (e.g., for MDRTB or simply to shorten those used for drug-sensitive TB) at modest cost.

## Data Availability

All data are reported in the paper or in Supplementary material.

## Supplementary Materials

Supporting information as described can be downloaded at: https://www.mdpi.com/article/10.3390/antibiotics11101274/s1. Figure S1: Luminescence detection at different time points for clofazimine in TB-infected PMBCs obtained from donor 2. Figure S2: Luminescence detection at different time points for nitazoxanide in TB-infected PMBCs obtained from donor 2. Figure S3: Luminescence detection at different time points for meropenem in TB-infected PMBCs obtained from donor 2. Figure S4: Luminescence detection at different time points for faropenem in TB-infected PMBCs obtained from donor 2. Figure S5: Luminescence detection at different time points for tebipenem in TB-infected PMBCs obtained from donor 2.

## References

[1] World Health Organization (2019). Global Tuberculosis Report 2019.

[2] Sharma D., Dhuriya Y.K., Deo N., Bisht D. (2017). Repurposing and Revival of the Drugs: A New Approach to Combat the Drug Resistant Tuberculosis. Front. Microbiol..

[3] Tezera L.T., Bielecka M.K., Chancellor A., Reichmann M.T., Basim A.l., Shammari B.A., Brace P., Batty A., Tocheva A., Jogai S. (2017). Dissection of the host-pathogen interaction in human tuberculosis using a bioengineered 3-dimensional model. eLife.

[4] Bielecka M.K., Tezera L.B., Zmijan R., Drobniewski F., Zhang X., Jayasinghe S., Elkington P. (2017). A Bioengineered Three Dimensional Cell Culture Platform Integrated with Microfluidics To Address Antimicrobial Resistance in Tuberculosis. mBio.

[5] Tezera L.B., Bielecka M.K., Elkington P.T. (2017). Bioelectrospray Methodology for Dissection of the Host-pathogen Interaction in Human Tuberculosis. Bio-Protocol.

[6] Moodley R., Godec T.R. (2016). Short-course treatment for multidrug-resistant tuberculosis: The STREAM trials. Eur. Respir. Rev..

[7] World Health Organization (2018). Rapid Communication: Key Changes to Treatment of Multidrug- and Rifampicin-Resistant Tuberculosis (Mdr/Rr-Tb).

[8] Chambers H.F., Turner J., Schecter G.F., Kawamura M., Hopewell P.C. (2005). Imipenem for treatment of tuberculosis in mice and humans. Antimicrob. Agents Chemother..

[9] De Carvalho L.P.S., Lin G., Jiang X., Nathan C. (2009). Nitazoxanide Kills Replicating and Nonreplicating *Mycobacterium tuberculosis* and Evades Resistance. J. Med. Chem..

[10] Shigyo K., Ocheretina O., Merveille Y.M., Johnson W.D., Pape J.W., Nathan C.F., Fitzgerald D.W. (2013). Efficacy of nitazoxanide against clinical isolates of *Mycobacterium tuberculosis*. Antimicrob. Agents Chemother..

[11] Castillo-Salazar M., Sánchez-Muñoz F., Springall Del Villar R., Navarrete-Vázquez G., Hernández-Diazcouder A., Mojica-Cardoso C., García-Jiménez S., Toledano-Jaimes C., Bernal-Fernández G. (2021). Nitazoxanide Exerts Immunomodulatory Effects on Peripheral Blood Mononuclear Cells from Type 2 Diabetes Patients. Biomolecules.

[12] Yuan S., Yin X., Meng X., Chan J.F.-W., Ye Z.-W., Riva L., Pache L., Chan C.C.-Y., Lai P.-M., Chan C.C.-S. (2021). Clofazimine broadly inhibits coronaviruses including SARS-CoV-2. Nature.

[13] Saravanan P., Dusthackeer V.N.A., Rajmani R.S., Mahizhaveni B., Nirmal C.R., Rajadas S.E., Bhardwaj N., Ponnuraja C., Bhaskar A., Hemanthkumar A.K. (2021). Discovery of a highly potent novel rifampicin analog by preparing a hybrid of the precursors of the antibiotic drugs rifampicin and clofazimine. Sci. Rep..

[14] Memar M.Y., Yekani M., Ghanbari H., Shahi S., Sharifi S., Maleki Dizaj S. (2020). Biocompatibility, cytotoxicity and antibacterial effects of meropenem-loaded mesoporous silica nanoparticles against carbapenem-resistant Enterobacteriaceae. Artif. Cells Nanomed. Biotechnol..

[15] Noufflard H., Berteaux S. (1958). Antituberculous activity of compound B-663. Ann. Inst. Pasteur..

[16] Dey T., Brigden G., Cox H., Shubber Z., Cooke G., Ford N. (2012). Outcomes of clofazimine for the treatment of drug-resistant tuberculosis: A systematic review and meta-analysis. J. Antimicrob. Chemother..

[17] Nunn A.J., Phillips P.P.J., Meredith S.K., Chiang C.-Y., Conradie F., Dalai D., Van Deun A., Dat P.-T., Lan N., Master I. (2019). A Trial of a Shorter Regimen for Rifampin-Resistant Tuberculosis. N. Engl. J. Med..

[18] Aung K.J., Van Deun A., Declercq E., Sarker M.R., Das P.K., Hossain M.A., Rieder H.L. (2014). Successful ‘9-month Bangladesh regimen’ for multidrug-resistant tuberculosis among over 500 consecutive patients. Int. J. Tuberc. Lung. Dis..

[19] Kaniga K., Cirillo D.M., Hoffner S., Ismail N.A., Kaur D., Lounis N., Metchock B., Pfyffer G.E., Venter A. (2016). A Multilaboratory, Multicountry Study To Determine MIC Quality Control Ranges for Phenotypic Drug Susceptibility Testing of Selected First-Line Antituberculosis Drugs, Second-Line Injectables, Fluoroquinolones, Clofazimine, and Linezolid. J. Clin. Microbiol..

[20] Pang Y., Zong Z., Huo F., Jing W., Ma Y., Dong L., Li Y., Zhao L., Fu Y., Huang H. (2017). Drug Susceptibility of Bedaquiline, Delamanid, Linezolid, Clofazimine, Moxifloxacin, and Gatifloxacin against Extensively Drug-Resistant Tuberculosis in Beijing, China. Antimicrob. Agents Chemother..

[21] Maurer F.P., Shubladze N., Kalmambetova G., Felker I., Kuchukhidze G., Koser C.U., Cirillo D.M., Drobniewski F., Yedilbayev A., Ehsani S. (2022). Diagnostic Capacities for Multidrug-Resistant Tuberculosis in the World Health Organization European Region: Action is Needed by all Member States. J. Mol. Diagn..

[22] Broekhuysen J., Stockis A., Lins R.L., De Graeve J., Rossignol J.F. (2000). Nitazoxanide: Pharmacokinetics and metabolism in man. Int. J. Clin. Pharmacol. Ther..

[23] Sotgiu G., D′ambrosio L., Centis R., Tiberi S., Esposito S., Dore S., Spanevello A., MigliorI G.B. (2016). Carbapenems to Treat Multidrug and Extensively Drug-Resistant Tuberculosis: A Systematic Review. Int. J. Mol. Sci..

[24] Gonzalo X., Satta G., Ortiz Canseco J., Mchugh T.D., Drobniewski F. (2020). Ertapenem and Faropenem against *Mycobacterium tuberculosis*: In vitro testing and comparison by macro and microdilution. BMC Microbiol..

[25] Gonzalo X., Drobniewski F. (2012). Is there a place for β-lactams in the treatment of multidrug-resistant/extensively drug-resistant tuberculosis? Synergy between meropenem and amoxicillin/clavulanate. J. Antimicrob. Chemother..

[26] Guo Z.Y., Zhao W.J., Zheng M.Q., Liu S., Yan C.X., Li P., Xu S.F. (2019). Activities of Biapenem against *Mycobacterium tuberculosis* in Macrophages and Mice. Biomed. Environ. Sci..

[27] Van Rijn S.P., Zuur M.A., Anthony R., Wilffert B., Van Altena R., Akkerman O.W., De Lange W.C.M., Van Der Werf T.S., Kosterink J.G.W., AlffenaaR J.-W.C. (2019). Evaluation of Carbapenems for Treatment of Multi- and Extensively Drug-Resistant *Mycobacterium tuberculosis*. Antimicrob. Agents Chemother..

[28] Reichmann M.T., Tezera L.B., Vallejo A.F., Vukmirovic M., Xiao R., Reynolds J., Jogai S., Wilson S., Marshall B., Jones M.G. (2021). Integrated transcriptomic analysis of human tuberculosis granulomas and a biomimetic model identifies therapeutic targets. J. Clin. Invest..

[29] Tezera L.B., Bielecka M.K., Ogongo P., Walker N.F., Ellis M., Garay-Baquero D.J., Thomas K., Reichmann M.T., Johnston D.A., Wilkinson K.A. (2020). Anti-PD-1 immunotherapy leads to tuberculosis reactivation via dysregulation of TNF-α. eLife.

[30] Andreu N., Zelmer A., Fletcher T., Elkington P.T., Ward T.H., Ripoli J., Parish T., Bancroft G.J., Schaible U., Robertson B.D. (2010). Optimisation of Bioluminescent Reporters for Use with Mycobacteria. PLoS ONE.

[31] Kothekar A., Divatia J.V., Myatra S.N., Gota V. (2020). Response to: 500 mg as bolus followed by an extended infusion of 1500 mg of meropenem every 8 h failed to achieve in one-third of the patients an optimal PK/PD against nonresistant strains of these organisms: Is CRRT responsible for this situation?. Ann. Intensive Care.

[32] Li Z., Su M., Cheng W., Xia J., Liu S., Liu R., Sun S., Feng L., Zhu X., Zhang X. (2021). Pharmacokinetics, Urinary Excretion, and Pharmaco-Metabolomic Study of Tebipenem Pivoxil Granules After Single Escalating Oral Dose in Healthy Chinese Volunteers. Front. Pharmacol..

[33] Gettig J.P., Crank C.W., Philbrick A.H. (2008). Faropenem medoxomil. Ann. Pharmacother..

[34] Abdelwahab M.T., Wasserman S., Brust J.C., Gandi N.R., Meintjes G., Everitt D., Diacon A., Dawson R., Wiesner L., Svensson E.M. (2020). Clofazimine pharmacokinetics in patients with TB: Dosing implications. J. Antimicrob. Chemother..

[35] Imani S., Buscher H., Marriott D., Gentili S., Sandaradura I. (2017). Too much of a good thing: A retrospective study of β-lactam concentration-toxicity relationships. J. Antimicrob. Chemother..

[36] Stockis A., Deroubaix X., Lins R., Jeanbaptise B., Calderon P., Rossignol J.F. (1996). Pharmacokinetics of nitazoxanide after single oral dose administration in 6 healthy volunteers. Int. J. Clin. Pharmacol. Ther..

