# Supplementary material for: Anti-Tuberculosis Activity of Three Carbapenems, Clofazimine and Nitazoxanide Using a Novel Ex Vivo Phenotypic Drug Susceptibility Model of Human Tuberculosis
Source: Antibiotics (Basel). 2022 Sep 20;11(10):1274. doi: 10.3390/antibiotics11101274 (PMC9598577; doi:10.3390/antibiotics11101274)
Supplement: Supplementary file 1 [file antibiotics-11-01274-s001.zip › antibiotics-1899873-supplementary-updated.pdf]

Supplementary figures

Supplementary Figure S1

**Luminescence detection at different time points for clofazimine in TB-infected PMBCs obtained from donor 2**

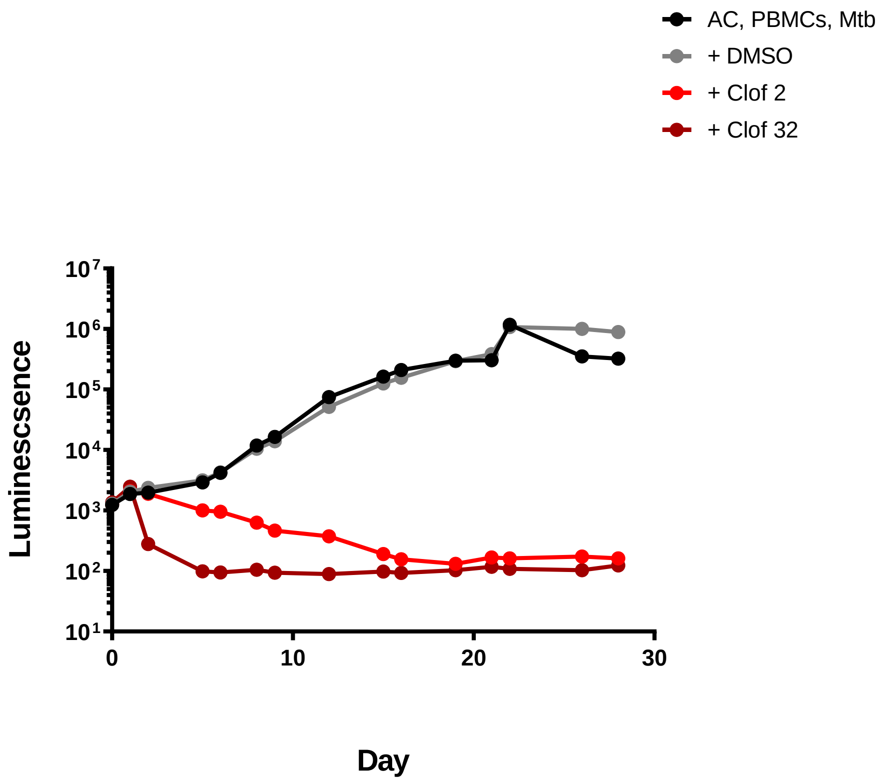


**AC: alginate collagen; PMBCs: peripheral blood mononuclear cells; Mtb: Mycobacterium tuberculosis; DMSO: dimethyl sulfoxide; Clof 2: Clofazimine 2mg/L; Clof 32: Clofazimine 32mg/L**

**Supplementary Figure S2: Luminescence detection at different time points for nitazoxanide in TB-infected PMBCs obtained from donor 2**

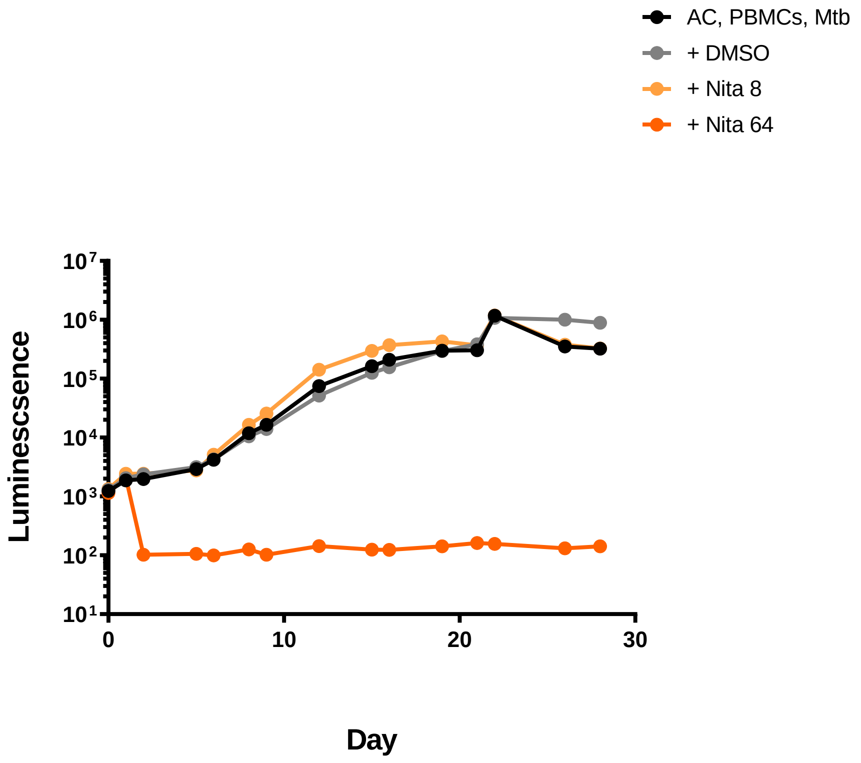


**AC: alginate collagen; PMBCs: peripheral blood mononuclear cells; Mtb: Mycobacterium tuberculosis; DMSO: dimethyl sulfoxide; Nita 8: Nitazoxanide 8mg/L; Nita 64: Nitazoxanide 64mg/L**

**Supplementary Figure S3: Luminescence detection at different time points for meropenem in TB-infected PMBCs obtained from donor 2**

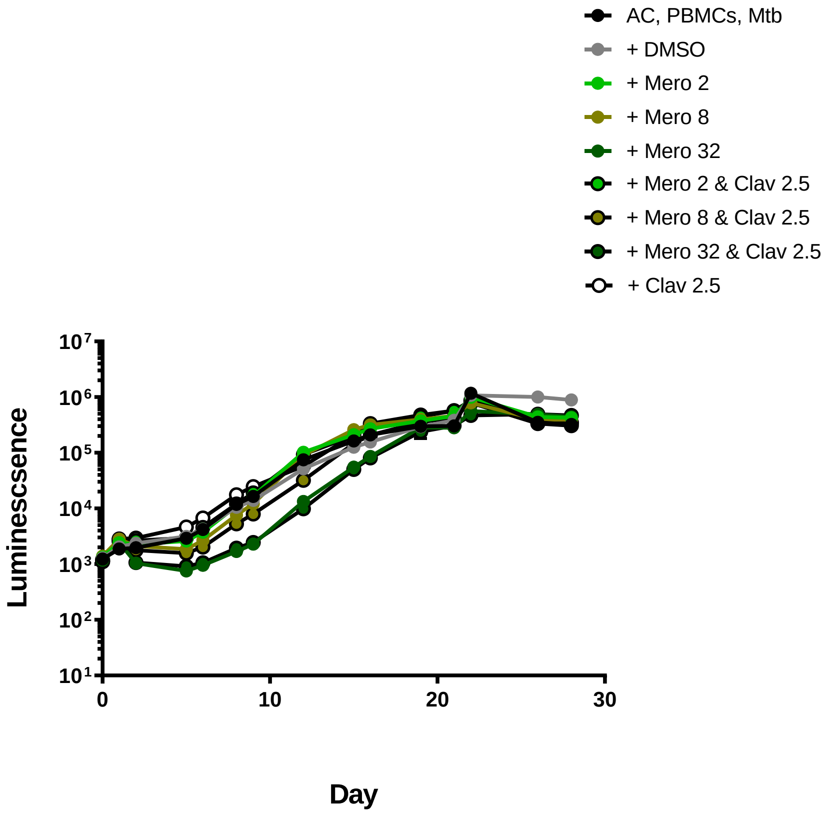


**AC: alginate collagen; PMBCs: peripheral blood mononuclear cells; Mtb: Mycobacterium tuberculosis; DMSO: dimethyl sulfoxide; Mero 2: Meropenem 2mg/L; Mero8: Meropenem 8mg/L; Mero 32: Meropenem 32mg/L; Clav 2.5: clavulanic acid 2.5 mg/L**

**Supplementary Figure S4: Luminescence detection at different time points for faropenem in TB-infected PMBCs obtained from donor 2**

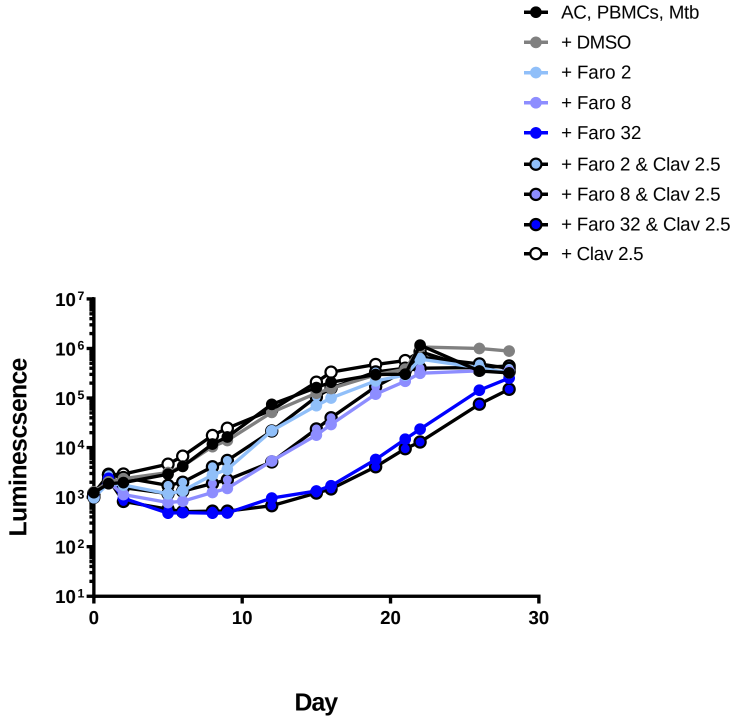


**AC: alginate collagen; PMBCs: peripheral blood mononuclear cells; Mtb: Mycobacterium tuberculosis; DMSO: dimethyl sulfoxide; Faro 2: Faropenem 2mg/L; Faro 8: Faropenem 8mg/L; Faro 32: Faropenem 32mg/L; Clav 2.5: clavulanic acid 2.5 mg/L**

**Supplementary Figure S5: Luminescence detection at different time points for tebipenem in TB-infected PMBCs obtained from donor 2**

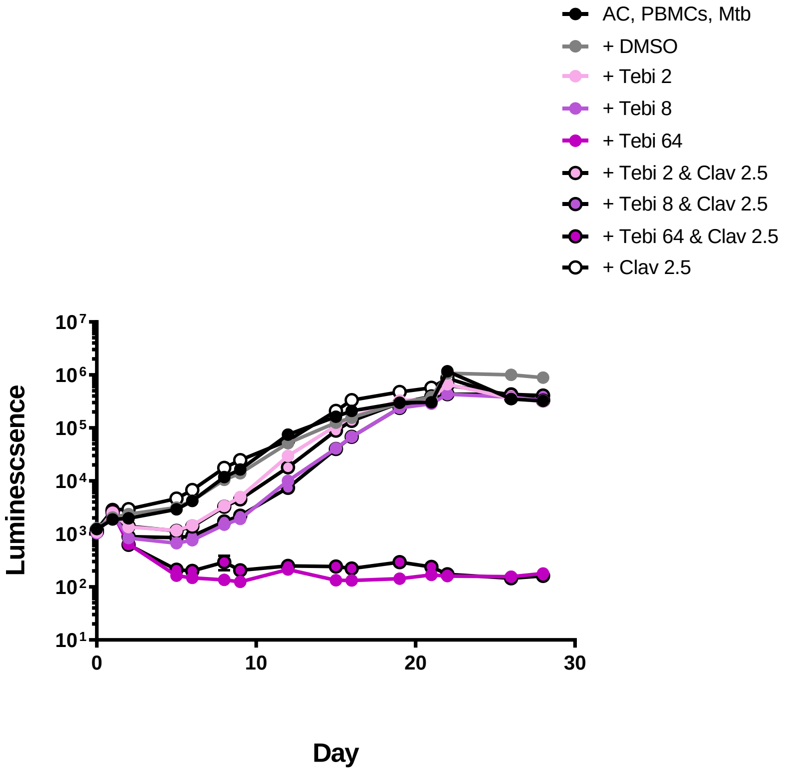


**AC: alginate collagen; PMBCs: peripheral blood mononuclear cells; Mtb: Mycobacterium tuberculosis; DMSO: dimethyl sulfoxide; Tebipenem 2: Tebipenem 2mg/L; Tebi 8: Tebipenem 8mg/L; Tebi 64: Tebipenem 64mg/L; Clav 2.5: clavulanic acid 2.5 mg/L**
